# Identifying palliative home care needs of patients with advanced cancer: a cross-sectional study

**DOI:** 10.3389/fpsyt.2025.1448973

**Published:** 2025-02-25

**Authors:** Xiaocheng Liu, Hui Liu, Wenjuan Ying, Yuying Zhang, Xiaoling Gong, Junfang Huang

**Affiliations:** ^1^ Pediatric Neurorehabilitation Department, Shenzhen Longhua Maternity and Child Healthcare Hospital, Shenzhen, China; ^2^ Oncology Department, Cancer Hospital of Shantou University Medical College, Shantou, China; ^3^ Nursing Department, The First Affiliated Hospital of Shantou University Medical College, Shantou, China

**Keywords:** palliative home care, home care, quality of life, need, cancer

## Abstract

**Background:**

Home-based palliative care is an ideal model for providing continuous, effective, and timely care at the patient’s home. However, the timely recognition of palliative home care needs remains a clinical challenge, and few studies have described the characteristics of palliative care needs and quality of life at home.

**Objectives:**

To identify the palliative home care needs of patients with advanced cancer and explore the influencing factors in addressing these needs.

**Methods:**

In this cross-sectional study, convenience samples were collected from four cities in mainland China between May 2020 and November 2021. The revised Problems and Needs in Palliative Care-short version was used to evaluate palliative home care needs. The European Organization for Research and Treatment of Cancer Quality of Life Questionnaire-C30 was used to evaluate the participants’ quality of life. We used SPSS version 23.0 for all statistical analyses. Descriptive statistics, Wilcoxon rank sum test, Spearman rho correlation independent t-test, and multiple linear regression were performed to analyze the data.

**Results:**

Completed the survey. The patients’ palliative home care needs were primarily related to physical symptoms, home health care services, and psychological issues. For items, the top three palliative home care needs were related to financial needs, “extra expenditures because of the disease” (75.9%), psychological issues, “fear of getting worse” (72.8%), and “fear of physical suffering” (67.9%). Multiple linear regression analysis revealed that 53.5% of palliative home care needs could be explained by the Karnofsky Performance Status and quality of life: physical, role, emotional, and financial difficulties dimensions.

**Conclusion:**

The palliative home care needs were associated with patients’ quality of life and financial difficulties. These findings may contribute to provide a framework for palliative home care service, and help to identify specific interventions.

## Introduction

Palliative care as an approach aimed at improving the quality of life of patients and their families experiencing a life-threatening illness (1). In home-based palliative care the patient is cared for in their home, while healthcare professional teams visit as required. The home is typically regarded as the preferred place of death for terminally ill patients. Palliative home care aims to deliver services common to most palliative care services, which conclude pain and symptom management, advanced care planning, psychosocial support, and caregiver support (1, 2). Expanding the scope of the palliative care model, palliative care for terminally ill patients is spreading rapidly from hospitals to homes, and in turn enable more patients to be cared for and to die at home (3). Palliative home care services can improve the quality of life, prolong survival and care satisfaction, reduce symptom burden, and improve health care use (4). Palliative home care has grown substantially the attention of practitioners, researchers and patients as well as families in recently years (5). Although the benefits and increasing demand for palliative home care have been demonstrated, challenges remain in providing optimal services (6).

Approximately 7.5 million people require palliative care each year, and yet, less than 1% of these individuals currently receive it in mainland China (7, 8). The 2021 Quality of Death and Dying ranks the quality of deaths globally, with Taiwan ranked 3^rd^, Hong Kong 9^th^, and mainland China 53^rd^ (9). This ranking reflects points out the urgency for improvements in China. Although policies and guidelines have been actively promoted in recent years, and palliative home care system has yet to be fully established (10). The transition of patients with terminally ill cancer receiving palliative care from hospital to home is a complex and challenging process. For cancer patients, long-term periods of experiencing cancer and anticancer treatments will bring numerous challenges and difficulties to patients and their families. Optimal palliative home care requires early identification of patients’ quality of life and supportive care needs (11, 12). A systematic review reported that the most frequently palliative needs was effective communication with health-care professional (13). Another study reported the palliative home care needs from the caregivers’ perspective, the caregivers expressed the need for more information on what palliative care, and how caring for a dying patient at home (14). Differences in cultural and social barriers impacting the provision of palliative care have been reported (15). Cultural beliefs about death and communication barriers are also challenges to palliative home care acceptance (16, 17). This leads to patients’ needs being ignored by palliative care facilities, resulting in unmet needs. Patients’ unmet needs or unsolved problems decrease their quality of life and harm their health outcomes (18).

Quality of life is defined as the quality of life affected by illness and is a multidimensional measure including physical, social, and emotional health (19). Studies have analyzed the relationship between palliative care and health-related quality of life in chronically ill individuals (2). However, few studies have examined this association in home-based palliative care, and the predictors of palliative home care needs are unclear. The objective of this study was to identify the palliative home care needs of patients with advanced cancer and explore the influencing factors in addressing these needs.

## Methods

### Participants and procedure

This cross-sectional study was conducted between May 2020 and November 2021 in four cities in mainland China. Participants were recruited if they were older than 18 years, diagnosed with stage II, III or IV cancer, in hospital-based palliative care or at home, and able to read and write in Chinese. Participants in hospital-based palliative care wards are assessed prior to the patient’s discharge home. Participants with dementia or severe cognitive impairment were excluded. All questionnaires were collected by three investigators, who used the same way to describe the purposes, contents, and potential benefits/risks of the study to all participants. All the participants provided written informed consent. Participants were invited to complete the questionnaires anonymously within 20 minutes. All questionnaires were retrieved after checking. This study was approved by the ethics committees of the First Affiliated Hospital of Shantou University Medical College (protocol number 2019088) and Shantou Longhu People’s Hospital (protocol number LHLL2020005).

### Demographic and clinical information

The self-reported demographic information included age, sex, education, religious beliefs, marital status, work status, residence, number of children, whether they lived alone, and health insurance status. Clinical information regarding the diagnosis, disease duration, cancer stage, metastasis, and Karnofsky Performance Status (KPS) score was obtained from the medical records.

### Problems and needs in palliative care-short version

The Problems and Needs in Palliative Care-short version (PNPC-sv) is a specific palliative care needs assessment measure for patients with cancer developed by Osse et al. (20) and has been validated in China by Wang et al. (21). After obtaining authorization from Osse and Wang, our team conducted interviews on the palliative home care needs and experiences of 15 patients with advanced cancer to supplement the relevant items (22) and further revise the PNPC-sv through expert consultation. The revised PNPC-sv has 36 items across seven domains: daily activities, physical symptoms, social issues, psychological issues, spiritual issues, financial problems, and home care services. The psychometric properties of the revised PNPC-sv have been well-tested in patients with cancer in China (23). Similar to the original questionnaire (20), the revised PNPC-sv consists of the problem and need-for-care sections. For these items, participants were asked about the degree to which they experienced a problem (Yes, Somewhat, No) and their need for professional support for this problem (Yes, more, As much as now, No). Items are rated on a three-point scale ranging from 0 (“No”) to 2 (“Yes”). Higher scores indicate more problems and care needs. This study only evaluated palliative home care needs. A need-for-care part was used, and Cronbach’s alpha for the current study was 0.926.

### European organization for research and treatment of cancer quality of life questionnaire-C30 scale

The European Organization for Research and Treatment of Cancer Quality of Life Questionnaire-C30 (EORTC QLQ-C30) consists of 30 items, including five functioning scales (physical, role, emotional, social, and cognitive), three symptom scales (fatigue, nausea/vomiting, and pain), a global status scale, and six single items (dyspnea, sleep disturbances, appetite loss, constipation, diarrhea, and financial difficulties). The two items of the global health status scale are scored on a seven-point scale ranging from 1, “very poor,” to 7, “very good.” All other items are scored on a four-point scale ranging from 1, “not at all,” to 4, “very much.” All scales and single items are linearly converted to a 0–100 scale. Higher scores on the functioning scales and global health status indicated a better health condition, whereas higher scores on the symptom scales and single items indicated a worse status. The psychometric properties of the Chinese version have been well examined (24), and Cronbach’s alpha for the current study was 0.904.

### Statistical analysis

All statistical analyses were performed using the SPSS version 23.0. Descriptive statistics are described as frequency, percentage, means, and standard deviations (SD). Normal distribution was assessed by the Kolmogorov-Smirnov test. The dependent variable was not normally distributed, Wilcoxon rank sum test was used. Spearman rho correlation was used among the KPS, quality of life, and palliative home care needs. Multiple linear regression analysis was used to examine the potential factors associated with palliative home care needs. All statistical analyses were two-sided, and statistical significance was set at p < 0.05.

## Results

### Sample characteristics

In total, 440 of the 500 initially eligible patients completed all questionnaires (response rate: 88%). 361 were recruited at home and 79 in the palliative care wards. The average age of the patients was 56.7 years (SD = 14.0, range: 19–93). Most were women (57.0%), with less than a high school education (66.4%), non-religious (65.7%), and married (89.8%). The average time since they received a cancer diagnosis was 23.3 months (SD = 32.0, range: 1–240). The sample characteristics are provided in **Table 1**. Participant’s palliative home care needs were negatively correlated with KPS (r = -0.606, p < 0.01). The analysis of the participants’ characteristics associated with the PNPC-sv indicated that patients aged 60 years or older (p = 0.003), with less than a high school education (p = 0.021), religious beliefs (p < 0.01), unemployed or retired (p = 0.002), more than three children (p = 0.013), and with metastasis (p < 0.01) had a higher need for palliative home care. Further details of the univariate analyses are presented in **Table 1**.

**Table 1:** Association of sociodemographic and clinical characteristics with palliative home care needs for patients with advanced cancer (n = 440).

Variables	Number (%)	M (P_25_, P_75_)	Z	P
Age				
< 60 years	246 (55.9)	24.9 (10.0, 37.0)	-2.945	0.003
≥ 60 years	194 (44.1)	29.2 (14.0, 41.3)		
Sex				
Female	251 (57.0)	27.3 (14.0, 38.0)	-0.795	0.427
Male	189 (43.0)	26.1 (10.0, 40.0)		
Education level				
Less than high school	292 (66.4)	27.9 (14.0, 40.0)	-2.316	0.021
High school or more	148 (33.6)	24.6 (10.0, 37.0)		
Religious beliefs				
Not religious	289 (65.7)	25.1 (10.0, 37.0)	-3.496	<0.001
Religious	151 (34.3)	30.0 (18.0, 41.0)		
Marital status				
Single	22 (5.0)	32.0 (18.5, 48.5)	8.184	0.042
Married	393 (89.3)	26.4 (12.0, 39.0)		
Divorced	11 (2.5)	19.8 (7.0, 32.0)		
Widowed	14 (3.2)	34.6 (21.8, 47.0)		
Residence area				
Suburban or rural	223 (50.7)	26.7 (11.0, 39.0)	-0.227	0.821
Urban	217 (49.3)	26.9 (13.0, 39.0)		
Living alone				
Yes	28 (6.4)	26.5 (14.5, 37.0)	-0.107	0.915
No	412 (93.6)	26.8 (12.0, 39.0)		
Employment status				
Employed	82 (18.6)	21.8 (7.8, 32.3)	13.818	0.001
Sick leave/unemployed	193 (43.9)	26.5 (13.0, 39.0)		
Retired	165 (37.5)	29.6 (14.0, 40.5)		
Family monthly income per capita (RMB)				
≤ 1500	75 (17.0)	28.4 (14.0, 40.0)	1.865	0.601
1500–4000	157 (35.7)	26.0 (13.0, 37.0)		
4000–6000	99 (22.5)	26.9 (10.0, 39.0)		
≥ 6000	109 (24.8)	26.8 (11.0, 41.0)		
Number of children				
< 3	323 (73.4)	25.6 (11.0, 39.0)	-2.248	0.025
≥ 3	117 (26.6)	30.0 (17.0, 42.0)		
Health insurance				
Citizen program	313 (71.1)	27.5 (13.0, 40.0)	-0.687	0.492
Rural program	127 (28.9)	26.5 (11.5, 39.0)		
Primary cancer site				
Nasopharynx	58 (13.2)	21.1 (10.8, 30.5)	28.635	<0.001
Esophagus	15 (3.4)	35.4 (25.0, 43.0)		
Stomach	16 (3.6)	32.8 (14.3, 49.5)		
Lung	123 (28.0)	30.8 (17.0, 42.0)		
Breast	28 (6.4)	19.4 (7.0, 29.3)		
Liver	21 (4.8)	27.5 (18.0, 39.5)		
Colon/rectum	75 (17.0)	26.9 (10.0, 39.0)		
Gynecological	38 (8.6)	26.8 (11.0, 41.0)		
Others	66 (15.0)	26.8 (11.0, 41.0)		
Time since confirmed diagnosis (Months)				
≤ 12	244 (55.4)	26.3 (11.0, 37.0)	0.823	0.663
12–36	120 (27.3)	27.5 (14.0, 40.0)		
≥ 36	76 (17.3)	27.4 (13.0, 43.0)		
Cancer stage				
II	16 (3.6)	17.8 (4.0, 31.3)	13.752	0.001
III	89 (20.2)	23.0 (9.0, 36.0)		
IV	335 (76.1)	28.2 (14.0, 40.0)		
Metastasis				
Yes	346 (78.6)	20.7 (8.0, 33.0)	-4.248	<0.001
No	94 (21.4)	28.4 (14.0, 40.0)		

Wilcoxon rank sum test was used.

### Palliative home care needs

Patients rated their palliative home care needs with 26.8 points (SD = 16.5, range: 1–72). For domains, the top three palliative home care needs were related to physical symptoms (mean = 5.39, SD = 4.07), home health care services (mean = 5.04, SD = 3.47), and psychological issues (mean = 4.47, SD = 3.32). For items, the top five palliative home care needs were related to financial needs “extra expenditures because of the disease” (334 of 440, 75.9%), psychological issues “fear of getting worse” (320 of 440, 72.8%), “fear of physical suffering” (299 of 440, 67.9%), financial needs “low proportion of medical insurance reimbursement” (296 of 440, 67.3%), and home health care service “insufficient information” (292 of 440, 66.3%). Details about the palliative home care needs are presented in **Table 2**.

**Table 2:** Palliative home care needs (n = 440).

Item	Do you want professional attention for this? (need-for-care)			
	No (1)	As much as now (2)	Yes, more (3)	(2) + (3)*
Daily activities				
Body care, washing, dressing, or using the toilet	301 (68.4%)	56 (12.7%)	83 (18.9%)	31.6%
Personal transportation (cycling, driving a car, using public transportation, etc.)	245 (55.7%)	74 (16.8%)	121 (27.5%)	44.3%
Doing light housework (tidying up, etc.)	266 (60.5%)	58 (13.2%)	116 (26.4%)	39.5%
Difficulties in performing the usual activities	256 (58.2%)	92 (20.9%)	92 (20.9%)	41.8%
Being dependent on others	238 (54.1%)	87 (19.8%)	115 (26.1%)	45.9%
Experiencing loss of control over ones life	254 (57.7%)	87 (19.8%)	99 (22.5%)	42.3%
Physical symptoms				
Pain	179 (40.7%)	115 (26.1%)	146 (33.2%)	59.3%
Fatigue	158 (35.9%)	141 (32.0%)	141 (32.0%)	64.0%
Sleeping problems	192 (43.6%)	143 (32.5%)	105 (23.9%)	56.4%
Shortness of breath	268 (60.9%)	91 (20.7%)	81 (18.4%)	39.1%
Cough	296 (67.3%)	80 (18.2%)	64 (14.5%)	32.7%
Nausea and vomiting	261 (59.3%)	86 (19.5%)	93 (21.1%)	40.7%
Constipation	242 (55.0%)	105 (23.9%)	93 (21.1%)	45.0%
Sexual dysfunction	335 (76.1%)	47 (10.7%)	58 (13.2%)	23.9%
Social issues				
Problems with family member relationships	364 (87.2%)	44 (10.0%)	32 (7.3%)	17.3%
Difficulties in talking about the disease with family members	322 (73.2%)	68 (15.5%)	50 (11.4%)	26.9%
Difficulties in talking about the disease, because of not wanting to burden others	241 (54.8%)	113 (25.7%)	86 (19.5%)	45.2%
Finding/feeling discriminated against by others because of illness	317 (72.0%)	78 (17.7%)	45 (10.2%)	27.9%
Difficulties in finding someone to talk to (confidant)	311 (70.7%)	64 (14.5%)	65 (14.8%)	29.3%
Psychological issues				
Depressed mood	232 (52.7%)	116 (26.4%)	92 (20.9%)	47.3%
Fear of physical suffering	141 (32.0%)	122 (27.7%)	177 (40.2%)	67.9%
Fear of getting worse	120 (27.3%)	112 (25.5%)	208 (47.3%)	72.8%
Difficulty coping with the unpredictability of the future	186 (42.3%)	130 (29.5%)	124 (28.2%)	57.7%
Difficulties in showing emotions	244 (55.5%)	106 (24.1%)	90 (20.5%)	44.6%
Spiritual issues				
Difficulties in accepting the disease	235 (53.4%)	102 (23.2%)	103 (23.4%)	46.6%
Difficulties in being available for others	206 (46.8%)	88 (20.0%)	146 (33.2%)	53.2%
Feeling like a burden to others	182 (41.4%)	131 (29.8%)	127 (28.9%)	58.7%
Financial problems				
Extra expenditures because of the disease	106 (24.1%)	93 (21.1%)	241 (54.8%)	75.9%
Loss of income because of the disease	158 (35.9%)	88 (20.0%)	194 (44.1%)	64.1%
Low proportion of medical insurance reimbursement	144 (32.7%)	114 (25.9%)	182 (41.4%)	67.3%
Home health care service				
Insufficient information, e.g., about the disease and its treatment, aids and agencies that can provide help, alternative healing methods, etc.	148 (33.6%)	159 (36.1%)	133 (30.2%)	66.3%
Home safety knowledge (fall prevention, falling out of bed, etc.)	252 (57.3%)	84 (19.1%)	104 (23.6%)	42.7%
Health education on pain medication	189 (43.0%)	96 (21.8%)	155 (35.2%)	57.0%
Diet instruction	170 (38.6%)	156 (35.5%)	114 (25.9%)	61.4%
Specialized care (drainage tube, PICC, infusion port, stress injury, wound or ostomy care, etc.)	208 (47.3%)	77 (17.5%)	155 (35.2%)	52.7%
Functional exercise	208 (47.3%)	140 (31.8%)	92 (20.9%)	52.7%

* (2) + (3) indicates the total percentage of patients who rated the item as either as much as as now or yes, more.

### Overall EORTC QLQ-C30

For the functional scales, the highest mean score was for cognitive functioning (mean = 71.6, SD = 25.8). The lowest mean score was for social functioning (54.1, SD = 29.5). In the symptom subscale, the highest mean score was for fatigue (mean = 46.3, SD = 25.1). The lowest mean score was for diarrhea (mean = 17.9, SD = 24.0). The global health/quality of life score was 54.5 (SD = 21.3). Details about the EORTC QLQ-C30 are presented in **Table 3**.

**Table 3:** Mean scores (M)/standard deviations (SD) of EORTC QLQ-C30 (n = 440).

Domain	M±SD
Function subscales	
Physical functioning	64.0 ± 31.6
Role functioning	58.5 ± 35.5
Emotional functioning	69.3 ± 23.4
Cognitive functioning	71.6 ± 25.8
Social functioning	54.1 ± 29.5
Symptom subscales/items	
Fatigue	46.3 ± 25.1
Nausea/vomiting	26.7 ± 28.6
Pain	35.0 ± 29.9
Dyspnea	22.8 ± 28.2
Insomnia	33.0 ± 29.3
Appetite loss	40.9 ± 30.6
Constipation	29.8 ± 29.6
Diarrhea	17.9 ± 24.0
Financial difficulties	46.3 ± 32.2
**Global health/Quality of life**	54.5 ± 21.3

M, mean.

SD, standard deviation.

EORTC QLQ-C30, The European Organization for Research and Treatment of Cancer Quality of Life Questionnaire-C30.

### Correlation analysis: palliative home care needs

Palliative home care needs were negatively correlated with physical functioning (r = -0.588, p < 0.01), role functioning (r = -0.575, p < 0.01), emotional functioning (r = -0.455, p < 0.01), cognitive functioning (r = -0.458, p < 0.01), social functioning (r = -0.541, p < 0.01), and global health/quality of life (r = -0.485, p < 0.01). Participants with a better quality of life had fewer palliative home care needs. Conversely, all symptom subscales were positively correlated with higher needs (r = 0.192 – 0.511, p < 0.01). Financial difficulties were positively correlated with palliative home care needs (r = 0.410, p < 0.01).

### Multiple linear regression analysis for palliative home care needs

Multiple linear regression analysis was performed to identify the factors influencing palliative home care needs. The total revised PNPC-sv score was the dependent variable. Significant variables in the previous univariate and correlation analyses (p < 0.05) were entered as independent variables. Multiple regression analyses (**Table 4**) indicated that the KPS, physical functioning, role functioning, and emotional functioning were negatively related to palliative home care needs. Patients who experienced nausea/vomiting, pain, sleep disturbances, and financial difficulties were positively correlated with palliative home care needs. It is worth noting that the model had high explanatory power, showing that 53.5% of the variance in palliative home care needs could be explained by the KPS and quality of life.

**Table 4:** Multiple linear regression analysis of factors associated with palliative home care needs (N = 440).

Factors	Unstandardized coefficients (B)	Standard error (SE)	Standardized coefficients (β)	P	95% CI
Constant	46.623	7.806	–	<0.01	31.278 to 61.968
KPS	-0.302	0.048	-0.367	<0.01	-0.395 to -0.208
Physical functioning	-0.079	0.039	-0.151	0.044	-0.156 to -0.002
Role functioning	-0.116	0.029	-0.250	<0.01	-0.173 to -0.059
Emotional functioning	-0.113	0.031	-0.160	<0.01	-0.174 to -0.051
Nausea/vomiting	0.059	0.025	0.103	0.016	0.011 to 0.107
Pain	0.049	0.025	0.089	0.048	0.000 to 0.098
Sleep disturbances	0.054	0.023	0.095	0.020	0.009 to 0.099
Financial difficulties	0.092	0.021	0.179	<0.01	0.050 to 0.133

CI, confidence interval; KPS: Karnofsky Performance Status.

B, unstandardized coefficients.

SE, standard error.

β: standardized coefficients.

R^2^ = 0.562, Adjust R^2^ = 0.535, F = 20.407, p < 0.01.

## Discussion

This is the first study to investigate the need for palliative care in Chinese patients with advanced cancer and examine the association between quality of life and individual characteristics of palliative care needs. Unsurprisingly, palliative home care needs are multifaceted and complex. Patients reported higher palliative home care needs when they had a poorer quality of life and vice versa. Additionally, multivariable analyses indicated a significant association between palliative home care needs and role functioning, emotional functioning, and financial difficulties. Identifying and understanding patients’ palliative home care needs and the influencing factors can provide health care providers with a theoretical basis for managing and treating patients with cancer at home. This is also a critical step toward determining palliative home care interventions.

Physical functioning is a crucial domain for a patient’s quality of life, and symptom control is an essential goal of palliative home care. Our findings indicate that the KPS score, physical functioning, and symptoms of nausea/vomiting, pain, and sleep disturbance were negatively correlated with palliative home care needs. In the current study, 64% of patients experienced fatigue, which was the most distressing and frequent symptom. Fatigue negatively affects daily functioning and health-related quality of life (25). Previous studies have reported that fatigue is a significant risk factor for falling (26, 27). This study reported that 42.7% of patients hoped to obtain more home safety knowledge (fall prevention, falling out of bed, etc.), which suggests that health care providers should pay attention to strengthening the assessment of fatigue symptoms and health education for fall prevention. Several patients described pain as the most universal symptom of discomfort, and they were afraid of opioid use because of fear of addiction and side effects (22). This study reported that 57% of the patients hoped to obtain more information about pain medications. This indicates that health care providers should help patients manage pain and provide knowledge about opioids.

Patients have frequently reported the need for home health care services. Among the highest-ranked items were insufficient information (e.g., about the disease and its treatment, aids and agencies that can provide help, and alternative healing methods), diet instruction, and health education on pain medications. These items indicate that patients need to obtain more illness-related and daily care-related information. Unmet home care information needs resulted in negative experiences for patients and caregivers (28). Conversely, sufficient information was associated with satisfaction, patient outcomes, and preference for palliative home care services. Given the importance of meeting patient information needs in palliative home care services, further research is needed to explore the effectiveness of various solutions to meet these needs.

Multivariable analysis indicated that financial difficulties were positively related to higher palliative home care needs. Financial difficulties were highly reported in this study, consistent with previous study (29). Financial difficulties were often reported by patients with cancer, in part due to work disruptions, income loss and high medical expenses. The current national health insurance does not shield patients and families from financial difficulties (30). Financial difficulties were associated with worse patient symptoms, poor quality of life and increased burden and mortality (29, 31), which was in turn increased palliative home care needs. These findings highlight the significance and need for routine assessment of financial problems in palliative home care services.

In models adjusted for demographic factors, we found an association between the overall quality of life and palliative home care needs. Physical symptoms such as fatigue, appetite loss, pain, and insomnia are common among patients at home and further challenge palliative home care management. Role functioning and emotional functioning were found to be associated with decreased quality of life, and higher palliative home care needs in patients with cancer. The diagnosis and side effects of anticancer treatment usually affect physical and mental health, resulting in role and emotional functioning disadvantages (32). Diminished role functioning may lead to disease burden and disengagement, limiting social and physical activities (33). Decreased emotional functioning can also develop along with anxiety, depression, and/or somatization, which may affect treatment tolerance and disease progression. Our results reinforce the need to consider patients’ perspectives on health care providers’ discussions of palliative home care interventions according to their needs and preferences.

This study provides a comprehensive understanding of the palliative home care needs and quality of life of patients with advanced cancer in China. However, the sample size was relatively small, and this population may not be representative of other provinces in China. Although we found a trend toward an association among the KPS, quality of life, and palliative home care needs, this study had a cross-sectional design; thus, causality cannot be fully confirmed. The study period was limited so the number of patients who passed away at home were not in a position to be observed. With the progression of the disease and the decline in quality of life, patients’ needs for palliative home care may change dynamically. Future longitudinal studies should consider changes in palliative home care needs throughout the illness rather than just focusing on a specific treatment period.

The current study can be the basis for further research and provide evidence to health care teams for tailored palliative home care interventions or service development. Palliative home care services should thoroughly evaluate the physical, psychological, and financial aspects to ensure the best possible quality of life for patients. Understanding the predictors of the need for palliative home care can assist health care teams in meeting the complex needs of patients. Evaluating and identifying these needs may contribute to implementing effective palliative home care services and interventions to improve patient and caregiver outcomes. Health care providers and policymakers should consider and address these factors to provide higher levels of care.

## Data Availability

The original contributions presented in the study are included in the article/Supplementary Material. Further inquiries can be directed to the corresponding author.
